# Supplementary material for: Smaller preferred interpersonal distance for joint versus parallel action
Source: PLoS One. 2023 May 2;18(5):e0285202. doi: 10.1371/journal.pone.0285202 (PMC10153701; doi:10.1371/journal.pone.0285202)
Supplement: S1 Table — Item numbers for Perceived Infectability dimension in bold. Item numbers for Germ Aversion dimension in italics. Reverse-coded items are indicated with an asterisk. (PDF) [file pone.0285202.s001.pdf]

S1 Table. Perceived Vulnerability to Disease scale items. Item numbers for Perceived Infectability dimension in bold. Item numbers for Germ Aversion dimension in italics. Reverse-coded items are indicated with an asterisk.

Item	Statement
1	It really bothers me when people sneeze without covering their mouths
**2**	If an illness is ‘going around’, I will get it
*3**	I am comfortable sharing a water bottle with a friend
4	I do not like to write with a pencil someone else has obviously chewed on
**5***	My past experiences make me believe I am not likely to get sick even when my friends are sick
**6**	I have a history of susceptibility to infectious disease
7	I prefer to wash my hands pretty soon after shaking someone’s hand
**8**	In general, I am very susceptible to colds, flu and other infectious diseases
9	I dislike wearing used clothes because you do not know what the last person who wore it was like
**10**	I am more likely than the people around me to catch an infectious disease
*11**	My hands do not feel dirty after touching money
**12***	I am unlikely to catch a cold, flu or other illness, even if it is ‘going around’
*13**	It does not make me anxious to be around sick people
**14***	My immune system protects me from most illnesses that other people get
15	I avoid using public telephones because of the risk that I may catch something from the previous user
