# Supplementary material for: Smaller preferred interpersonal distance for joint versus parallel action
Source: PLoS One. 2023 May 2;18(5):e0285202. doi: 10.1371/journal.pone.0285202 (PMC10153701; doi:10.1371/journal.pone.0285202)
Supplement: S2 Table — (PDF) [file pone.0285202.s002.pdf]

S2 Table. Multiple regression analysis for *diffIPD* (Experiment 1).

	Unstandardised estimate	Standard error	β	t	p
Intercept	0.212	0.0861		2.46	.0146
Country	0.0187	0.0396	.0329	0.472	.637
Perceived Infectability	0.00934	0.0177	.0390	0.527	.599
Germ Aversion	0.00260	0.0209	.00957	0.124	.901
COVID-context awareness	-0.0173	0.0101	-.125	-1.72	.0867
