# Supplementary material for: Smaller preferred interpersonal distance for joint versus parallel action
Source: PLoS One. 2023 May 2;18(5):e0285202. doi: 10.1371/journal.pone.0285202 (PMC10153701; doi:10.1371/journal.pone.0285202)
Supplement: S3 Table — (PDF) [file pone.0285202.s003.pdf]

S3 Table. Multiple regression analysis for *diffIPD* (Experiment 2).

	Unstandardised estimate	Standard error	β	t	p
Intercept	0.129	0.0731		1.77	.0781
Country	0.0407	0.0346	.0822	1.18	.241
Perceived Infectability	0.00660	0.0160	.0303	0.413	.680
Germ Aversion	-0.00584	0.0173	-.0255	-0.337	.736
COVID-context awareness	-0.0111	0.00879	-.0913	-1.27	.207
