# Supplementary material for: Smaller preferred interpersonal distance for joint versus parallel action
Source: PLoS One. 2023 May 2;18(5):e0285202. doi: 10.1371/journal.pone.0285202 (PMC10153701; doi:10.1371/journal.pone.0285202)
Supplement: Attachment — Submitted filename: SchmitzReader2022_ReviewResponse.docx [file pone.0285202.s004.docx]

*Thank you for submitting your manuscript to PLOS ONE. We have now received the feedback from two independent reviewers, which are both positive overall. As you will see, Reviewer 2 listed a few minor concerns which I urge you to address.*

**Response:** We’d like to thank you and the two reviewers for volunteering your time to evaluate our work. We have made some suggested changes and provide point-by-point responses below.

*- I particularly agree with Reviewer 2 regarding clarity of the approach to joint/parallel action and aims of your study. For example, in H1 you used moving in synchrony in the exercise scenario as a prime for joint action whereas in H2 you used moving in synchrony in an exercise scenario as a prime for parallel action. We tend to adjust our movements to synchronise those of others automatically and moving in synchrony is inherently enjoyable (e.g., mirroring). Thus, with the goal of enjoyment, I would argue that if done consciously, moving in synchrony is a joint action. I think further elaboration here and in regards to the novelty of your study as pointed out by Reviewer 2 would strengthen your work.*

**Response:** We completely agree that moving in synchrony (if done consciously) constitutes a joint action, and we believe that our manuscript is consistent with that idea. Accordingly, we acknowledge the significance of synchrony in the General Discussion of our original manuscript as follows (see lines 579-588):

“It is also worth noting that in Experiment 2, two of the joint action scenarios (Exercise Class and Drumming lesson) contained an additional element of coordination, namely interpersonal synchrony. This was due to the rhythmic nature of the performed actions, stepping and drumming, respectively. Since previous research has shown that synchronous behaviour fosters prosocial behaviour, bonding, and affiliation between individuals (e.g., (53–55); for a meta-analysis, see (56)), we cannot exclude the possibility that the synchronous nature of the imagined joint actions might have had an additional effect on participants’ IPD preference. A systematic comparison between non-synchronous and synchronous joint action scenarios is needed to determine to what extent, if at all, interpersonal synchrony plays a role in individuals’ IPD preferences.”

Moreover, regarding the two exercise scenarios in Experiments 1 and 2, we believe that there might have been a misunderstanding which we’d like to resolve hereby. In Exp. 1, the joint action scenario (passing a ball back and forth) does not involve synchrony; neither does the parallel action (lifting a ball up and down individually). In the latter, parallel action, there is no synchronous movement requirement; individuals are free to (imagine that they) lift the ball at their own pace. In Exp. 2, the joint action (performing the same step routine in synchrony) is synchronous, whereas the parallel action (performing different step routines subsequently) is not.

*- In addition to the rating scale comments of Reviewer 2; I am concerned that your visuals are not context sensitive enough. As shown in your Figure 1, participants have to mentally translate a distance from the left to right to a figure to their own personal space. The paper you cited that used a similar approach is quite old (1973). Other more recent studies used more interactive approaches, including the other citation from 2016, which showed differences between active (moving) and passive (still) contexts. Would you expect that an active task using a 3D visualisation to be more sensitive than your IPD rating scale (that has considerably low ecological validity)?*

**Response:** We completely agree that active tasks with a 3D visualisation would be more ecologically valid and that future studies should strive to incorporate those. When designing our study, we built on recent studies investigating IPD preferences (e.g., Welsch et al., 2021; Sorokowska et al., 2017) that used the same methodology (i.e., a static 2D image of a person). Also, as we note in our discussion, effects observed using the sort of IPD rating scale as in our study tend to align well with effects observed using more naturalistic methods (Iachini et al., 2016). Most importantly though, in our study, we are investigating potential between-condition effects. If these effects exist (as our data suggest), they cannot be explained by the methodology we used, as this was the same across conditions.

*- My final concern was not highlighted by the reviewers and is regarding your analyses. Why did you run so many separate analyses with different calculated dependent measures (e.g., meanIPD)? I understand that you have pre-registered your approach, however, I understand that this was not peer-reviewed. I also do not understand why you report an impact of Covid related concerns in the discussion when the null model without them was clearly fitting the data better.*

**Response:** In addition to examining differences between Joint and Parallel action on preferred IPD, we assess the impact of four factors (Country, Perceived Infectability, Germ Aversion, and COVID-context awareness) on preferred IPD in two ways using regression analyses. MeanIPD is the mean of all IPD ratings, and provides a measure of individuals’ preferred IPD regardless of the social context. This is where we find an effect of COVID-related concerns, in keeping with previous research. We also examined the impact of the aforementioned factors on ‘diffIPD’, which is the difference between IPD ratings in the Parallel and Joint scenarios. The aim of this analysis was to reveal, for example, whether preferred IPD was more strongly influenced by the aforementioned factors in Joint than Parallel scenarios. We do not find any effect here, and it is correct to say that the Bayesian analysis reveals that the data were most likely under the null model. We describe the different approaches in the methods section, lines 248-274. The Bayesian analysis is post hoc in response to the non-significant regression for diffIPD (lines 310-318). We also distinguish between these results in the discussion, lines 507-517.

*Therefore, we invite you to submit a revised version of the manuscript that addresses all the points raised during this reviewing cycle.*

**Response:** Please find our point-to-point replies to the reviewers’ comments below. All changes in the manuscript have been marked in blue.

*Reviewers' comments:*

***Reviewer #1:***

*This study used surveys to study interpersonal distance preference during different type of social scenarios, namely joint actions where people are interacting towards a shared goal and parallel actions that may share a similar motor context but with no collaborative intent.*

*This paper is extremely well written. The hypotheses are clearly exposed, the methods are sound, and the authors are in line with open-science and reproducible science practices (pre-registration, preprint, power analysis, raincloud plots).*

*The authors predicted smaller preferred IDP for joint actions compared to parallel ones, and their empirical data clearly validate this prediction. Although this is not surprising per se, it is an important result to add to the field. It also validates online surveys as a good tool to inquire about socio-cognitive processes.*

*I congratulate the authors for doing such a good job and I thank them for making my job as a reviewer very easy.*

**Response:** Thank you very much for this positive evaluation of our work.

***Reviewer #2:***

*The authors tested whether the nature of the social interaction (joint vs parallel) and concerns about pathogen infection affect preferred interpersonal distance (IPD). The results showed that participants would prefer shorter IPD to perform joint as compared to parallel actions. This difference was not affected by concerns about pathogens but the overall preferred IPD (averaged over the two types of interaction) was, with people aversive to germs preferring larger IPD. The methods are particularly sound, e.g., large sample size (N = 200), replication experiment to control for the potential methodological issues of the first experiment, and pre-registration. I only have one relatively major concern and several minor concerns:*

**Response:** Thank you for the helpful feedback on our manuscript.

*- The original contribution of this study compared to previous studies is not sufficiently clear. Previous studies already showed an effect of collaboration vs. competitive tasks on preferred IPD. The additional contribution of studying joint vs. parallel actions should be developed as soon as in the introduction.*

**Response:** We believe that we have already addressed the additional contribution of studying joint action in the introduction. For example, after highlighting the factors that influence IPD (including cooperation/competition), we state that we wished to understand the influence of other distinct social interaction types. Ultimately, we consider this work a preliminary investigation - the exact reasons *why* different forms of social interaction would alter IPD require further study (as stated in the discussion). However, we have slightly expanded the rationale in lines 66-68:

“Understanding how personal space preferences change in these common forms of social behaviour can provide a more detailed picture of typical human interactions.”

*- Moreover, it has already been shown that Croat participants with germ aversion prefer to keep a larger IPD during the first months of the pandemic (Hromatko et al., 2021). More than two years later germ aversion has been found to have no direct effect on the French people’s IPD, but to modulate the effect of face masks on IPD, with people highly aversive to germs preferring confederates without face mask to be put further away (Geers & Coello, 2022). As for the current paper, only Germ Aversion (and not Perceived Vulnerability) had an effect. The author might cite these results in the introduction and discuss them in relation to theirs.*

**Response:** Thank you for highlighting these articles. We were not aware of them when planning and preregistering this work, and so it seems inappropriate to engage with them in the introduction as if this was the case. However, we have added Hromatko et al. (2021) to the method section where we discuss the possibility of Germ Aversion predicting IPD (line 269). We also added both citations to the discussion (lines 603-606).

*Minor:*

*- The example of joint vs. parallel actions in the introduction might not be the most relevant since it confounds the nature of the social interaction with the nature of the relation between the two confederates.*

**Response:** Thanks for pointing this out; we agree that the relation between the two agents (colleagues vs. strangers) constitutes a confound in our example. We adapted the example accordingly; see lines 54-68:

“Imagine, for instance, volunteers from a local community fixing recent storm damage by removing a fallen tree from a pathway. Two volunteers are operating a two-man saw (each holding one end of the saw) to cut a branch of the tree in half. Two other volunteers each operate their own saw to cut different branches of the tree. Comparing these two scenarios, we would consider the former two volunteers to be acting jointly and the latter two volunteers to be acting in parallel. Note that these two scenarios are merely meant to serve as intuitive examples illustrating the distinction between joint and parallel action – they are not meant to provide a complete definition. Instead, in line with a recent, comprehensive account (24), we assume that what differentiates a joint from a parallel action is that when individuals act jointly, their actions are collectively directed towards the same goal, such as cutting the same branch (e.g., (27); on collective/joint/shared goals, see (24,28,29)). Here, we asked whether the distinction between joint and parallel action might also be reflected in the distance individuals prefer to keep from one another during interaction. Understanding how personal space preferences change in these common forms of social behaviour can provide a more detailed picture of typical human interactions.”

We also provide some further consideration of varied ways in which one might engage in joint action in our discussion (lines 546-548), as well as the challenges of closely matching joint and parallel scenarios (lines 622-631).

*- The reliability of the Visual Analog Scale might already be presented in the Methods to justify their choice.*

**Response:** We have followed the reviewer’s suggestion and added this piece of information to the Methods section (lines 163-164).

*- Was the actual size of the rating scale on the participant’s screen controlled for? Position judgments depend on the physical length of the scale they have to be made on. For instance, the middle of a line is positioned towards the right vs. left as compared to the actual middle for short vs. long lines (McCourt & Jewell, 1999). So, performing the IPD rating on the screen of a smartphone vs. computer might induce some differences (and thus noise here) related to the size of the scale.*

**Response:** All participants completed the survey on their personal computers; participation on a smartphone was not possible. The size of the image was 996 x 472 pixels, which is dependent on participant monitor size. This has now been clarified in text (lines 165-167). Please note that this does not influence the comparison between the two conditions in our study because image size would always be the same within one participant.

*- The plot of the individual data is not very legible. The authors might consider decreasing the size of the dots*.

**Response:** Unfortunately this is not possible in JASP, but we do report the percentage of participants who show effects in each direction, providing comparable information.

*- I would announce the objectives of Experiment 2 in the introduction. The issues of the scenarios in Experiment 1 personally disturbed me until reading they would be addressed in Experiment 2.*

**Response:** We followed the reviewer’s suggestion and have added the objectives of Experiment 2 at the end of the Introduction, lines 105-108:

“We tested our hypotheses in two experiments, with the second performed to control for a possible confound in the first. This confound was related to the use of shared objects in joint action scenarios (see Experiment 1 discussion).”

*- Could the authors provide the corrections they made to the scenarios in Experiment 2?*

**Response:** For both Exp. 1 and Exp. 2, we provide the exact wording of all scenarios in Table 1. This has been clarified in the table title.

*- Another potential reason for the difference between the Storm Damage scenario and the other scenarios in Experiment 2, is that it requires manipulating a saw, i.e., a dangerous object that one generally avoids manipulating in the vicinity of someone.*

**Response:** The reviewer makes a good point, and we have addressed this in Experiment 2 discussion, lines 483-485:

“Alternatively, the comparable IPD ratings for the two versions could reflect the use of a potentially dangerous tool, such that individuals might maintain a more consistent distance for safety reasons, possibly overriding personal comfort.”
